# Efficient TGF-β/SMAD signaling in human melanoma cells associated with high c-SKI/SnoN expression

**DOI:** 10.1186/1476-4598-10-2

**Published:** 2011-01-06

**Authors:** Delphine Javelaud, Leon van Kempen, Vasileia I Alexaki, Erwan Le Scolan, Kunxin Luo, Alain Mauviel

**Affiliations:** 1 Institut Curie, University Center, Building 110, Orsay, France; 2 INSERM U1021, University Center, Building 110, Orsay, France; 3 CNRS UMR3347, University Center, Building 110, Orsay, France; 4 Université Paris XI, Orsay, France; 5 Radboud University Nijmegen Medical Centre, The Netherlands; 6 Department of Molecular and Cell Biology, University of California Berkeley, Berkeley, CA, USA

## Abstract

**Background:**

SKI and SnoN proteins have been shown to inhibit TGF-β signaling, acting both as transcriptional co-repressors in the cell nucleus, and as sequestrators of SMAD proteins in the cytoplasm. TGF-β, on the other hand, induces rapid, proteasome-mediated, degradation of both proteins. How elevated SKI and SnoN protein levels co-exist with active autocrine TGF-β signaling in cancer cells is yet to be understood.

**Results:**

In this study, we found elevated SKI and SnoN protein levels in a panel of melanoma cell lines, as compared to normal melanocytes. There was no correlation between SKI protein content and the capacity of melanoma cells to invade Matrigel™, to form subcutaneous tumors, or to metastasize to bone after intracardiac inoculation into nude mice. Nor did we find a correlation between SKI expression and histopathological staging of human melanoma. TGF-β induced a rapid and dose-dependent degradation of SKI protein, associated with SMAD3/4 specific transcriptional response and induction of pro-metastatic target genes, partially prevented by pharmacologic blockade of proteasome activity. SKI knockdown in 1205Lu melanoma cells did not alter their invasive capacity or transcriptional responses to TGF-β, and did not allow *p21 *expression in response to TGF-β or reveal any growth inhibitory activity of TGF-β.

**Conclusions:**

Despite high expression in melanoma cells, the role of SKI in melanoma remains elusive: SKI does not efficiently interfere with the pro-oncogenic activities of TGF-β, unless stabilized by proteasome blockade. Its highly labile nature makes it an unlikely target for therapeutic intervention.

## Background

Transforming growth factor-β (TGF-β) superfamily members signal via membrane-bound heteromeric serine-threonine kinase receptor complexes. Upon ligand binding, receptor activation leads to phosphorylation of cytoplasmic protein substrates of the SMAD family and subsequent accumulation in the nucleus where they act as transcription factors to regulate target gene expression [1-3]. TGF-β acts as a tumor suppressor by promoting cell cycle arrest or apoptosis of normal epithelial cells during early stages of carcinogenesis, while at later stages of tumorigenesis, it functions as a tumor promoter, inducing neoplastic cell invasiveness and metastasis through a process referred to as epithelial to mesenchymal transdifferentiation (EMT), and via modulation of the extracellular tumor microenvironment, production of chemokines and recruitment of immature bone marrow-derived myeloid cells to the invasive front of tumors, and inhibition of anti-tumoral immune defenses [4-8].

Members of the SKI family of proto-oncoproteins are involved in regulation of cellular transformation and differentiation [9]. SKI was originally identified as the transforming protein (v-ski) of the avian Sloan-Kettering virus, whose overexpression promotes anchorage-independent growth of chicken and quail embryo fibroblasts [10]. SKI (and SnoN) proteins are also important negative regulators of the TGF-β signaling cascade [11-13]. In the nucleus, SKI proteins repress SMAD ability to transactivate TGF-β target genes by disrupting active heteromeric complexes of SMAD2 or SMAD3 with SMAD4, by recruiting a transcriptional repressor complex containing N-CoR SMRT, Sin3A, and HDAC-1, and by blocking the binding of transcriptional coactivators [14-16]. SKI may also localize in the cytoplasm of tumor cells [17], where it may interfere with TGF-β signaling by sequestering SMAD proteins and preventing their nuclear accumulation in response to TGF-β, as we demonstrated in the case of SnoN [18]. The ability of SnoN and SKI to antagonize TGF-β-induced growth arrest is thought to be important for their transforming activity [19]. Inversely, other reports have shown cell-type specific effects of SnoN as a mediator of TGF-β signaling [20], and identified ING2 as a mediator of SnoN effects to promote TGF-β-driven transcription [21], thereby emphasizing the complexity of the interaction between SKI family members and TGF-β signaling. Furthermore, expression levels of SKI family members may be downregulated by TGF-β, as the latter rapidly induces SKI protein poly-ubiquitination and degradation in a SMAD- and proteasome-dependent manner, allowing TGF-β target gene transactivation [22-29].

Consistent with a potential oncogenic role, SKI and SnoN are often expressed at high levels in various human cancers cells derived from melanoma, esophageal cancer, pancreatic cancer and leukemia, due to increased transcription, gene amplification, and/or protein stabilization. Yet, SKI may also exert anti-tumorigenic activities: for example, *Ski+/- *mice display an increased susceptibility to chemical-induced tumorigenesis [30]. The human *SKI *gene is located at chromosome 1p36, a potential tumor suppressor locus that is frequently deleted in various human cancers including neuroblastoma, melanoma, colorectal carcinoma and leukemia [31]. Clearly, the roles of SKI in mammalian tumorigenesis are complex, and more studies are needed in order to define the functions of SKI.

Melanoma cells secrete large amounts of TGF-β: expression of TGF-β1 and β2 is increased in parallel with tumor stage, and all isoforms are expressed in highly aggressive melanoma [32-34]. In melanoma cells, constitutive SMAD signaling occurs in response to autocrine TGF-β secretion [35], and experimental blockade of TGF-β signaling by SMAD7 overexpression dramatically reduces their tumorigenic and metastatic potential [36,37]. Likewise, systemic pharmacologic inhibition of TGF-β signaling in mice prevents experimental melanoma cell metastasis to bone [38]. Remarkably, it has been reported that melanoma cells express high amounts of SKI protein, which localizes both in the nucleus and in the cytoplasm [17]. It has been suggested that such high expression of SKI blocks TGF-β transcriptional responses, in particular the induction of p21/WAF, resulting in an inactive TGF-β pathway in melanoma cells and lack of growth inhibitory activity of TGF-β [39,40]. SnoN may exert similar functions when SKI is not expressed in some melanoma cell lines [41]. It is widely accepted that TGF-β is a potent inducer of SKI (and SnoN) degradation [22-29], and we recently demonstrated that in breast cancer cells, TGF-β suppresses the ability of SKI to inhibit tumor metastasis by inducing its degradation via the ubiquitin-proteasome pathway, whereby TGF-β induces the E3 ubiquitin-ligase Arkadia to mediate SKI degradation in a SMAD-dependent manner [22].

We report that despite high levels of SKI protein expression, melanoma cells exhibit strong transcriptional responses to TGF-β. We provide definitive evidence for rapid and efficient dose-dependent degradation of SKI protein in response to exogenous TGF-β, through the ubiquitin-dependent proteasome pathway. Remarkably, SKI antagonism against TGF-β activity primarily occurred when SKI degradation in response to TGF-β was prevented by proteasome blockade. We also report that SKI levels do not correlate with the tumorigenic or metastatic potential of melanoma cells, the latter largely depending upon constitutive TGF-β signaling, and do not correlate with the clinical or pathological stage of human melanoma lesions.

## Results

### High SKI protein levels in human melanoma cell lines - Absence of correlation with Matrigel™ invasiveness, tumorigenicity or metastatic potential in vivo

**Figure 1:**
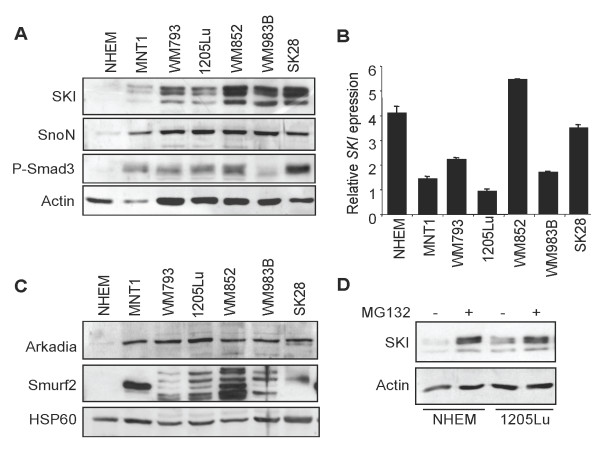
**SKI and SnoN expression in human melanoma cell lines**. A. Total protein extracts (40 μg) from unstimulated cultured melanoma cell lines and normal melanocytes were analyzed by Western blotting for SKI, SnoN and P-SMAD3 levels. An anti-actin antibody served for normalization. B. *SKI *mRNA steady-state levels, as measured by quantitative RT-PCR. C. Western analysis of the ubiquitin E3 ligases Arkadia and Smurf2 levels in the same cell extracts as in panel A. D. 1205Lu at approx. 80% confluency were incubated for 8 h with MG132 (10 μM) before protein extracts were prepared from cell lysates and processed for Western analysis of SKI content.

We first used Western analysis to evaluate SKI and SnoN protein levels in a panel of human melanoma cell lines as compared to normal melanocytes. As shown in Figure 1A, SKI and SnoN protein levels were barely detectable in normal melanocytes. On the other hand, all melanoma cell lines tested (WM793, 1205Lu, WM852, WM983B and SK28) expressed high levels of SKI and SnoN protein (Figure 1A). The non-tumorigenic MNT1 cell line expressed relatively similar levels of SKI protein, after correction for β-actin content, as compared to other melanoma cell lines with tumorigenic potential. Additional cell lines (Dauv-1, Fo-1, WM239A, WM1341D, SK-mel501, SK-mel888) exhibited similar high SKI protein content (not shown). These data are consistent with previous report on the subject [17]. P-SMAD3, a marker of constitutive TGF-β receptor activity, was detected in all melanoma cell lines that we examined, not in normal melanocytes, consistent with our initial observations of autocrine SMAD signaling in various human melanoma cell lines in culture [35]. *SKI *mRNA levels, as measured using quantitative RT-PCR (Figure 1B) were highly variable across melanoma cell lines, not higher than in normal melanocytes, and did not correlate with SKI protein levels, suggesting uncoupling of gene transcription and protein expression. Similar results were found for *SnoN *mRNA levels (not shown). Together, these data are consistent with the literature that describes SKI and SnoN proteins as targets for proteasomal degradation in response to TGF-β [22-29].

We next examined the expression of the ubiquitin ligases Arkadia and Smurf2, as these proteins are essential for proteasome-mediated degradation of SKI and SnoN proteins. As shown in Figure 1C, all melanoma cell lines exhibited elevated and rather similar levels of Arkadia and variable levels of Smurf2. Arkadia was hardly detectable in normal melanocytes, in which no expression of Smurf2 was found. Remarkably, treatment of normal melanocytes with the proteasome inhibitor MG132 allowed for a dramatic recovery of SKI protein levels (Figure 1D). MG132 treatment of 1205Lu melanoma cells treated resulted in increased SKI protein content, consistent with a role of the proteasome in controlling SKI protein levels, both in normal and malignant melanocytes.

Given our extensive phenotypic characterization of various melanoma cell lines using Matrigel™ invasion *in vitro *as well as subcutaneous tumor growth and bone metastasis in nude mice [36,42], we thought to determine whether basal SKI protein levels in culture may be predictive of a given invasive, tumorigenic, or metastatic behavior of melanoma cells. As shown in Table 1, SKI protein levels did not correlate with the capacity of melanoma cells to invade Matrigel™. Neither did they correlate with their capacity to form subcutaneous tumors in nude mice or with the incidence of bone metastasis following intracardiac inoculation of tumor cells into nude mice. Remarkably, all of these cellular activities are efficiently altered upon TGF-β inhibition by either SMAD7 overexpression or pharmacologic inhibitors of TβRI kinase activity *in vitro *or *in vivo *[36-38], attesting for pro-tumorigenic and pro-metastatic activities of autocrine TGF-β signaling despite high SKI and SnoN protein levels.

**Table 1:** No correlation between SKI levels and melanoma cell invasion, tumor growth and metastasis in mice.

Cell line	SKI protein levels	Matrigel™ invasion	Subcutaneous tumor growth in mice	Bone metastases in mice
SK28	100	8	90	40%

888mel	68	5	0	62.5

501mel	100	10	0	71.5

WM852	85	90	N.D.	100

1205Lu	65	100	95	100

Dauv1	53	20	50	100

Relative SKI protein levels in a series of cultured melanoma cell lines were determined by scanning densitometry of Western analyses. Values for subcutaneous tumor growth in mice represent tumor incidence at 40 days post-injection. Matrigel™ invasion results are presented as relative values compared to the 1205Lu cell line to which a value of 100 was given arbitrarily. Values for bone metastases in mice represent the incidence at 5 weeks post-inoculation of tumor cells. Details about experimental procedures may be found in [36,37]. N.D.: not determined

### TGF-β signaling is a critical determinant of SKI protein levels in melanoma cells

**Figure 2:**
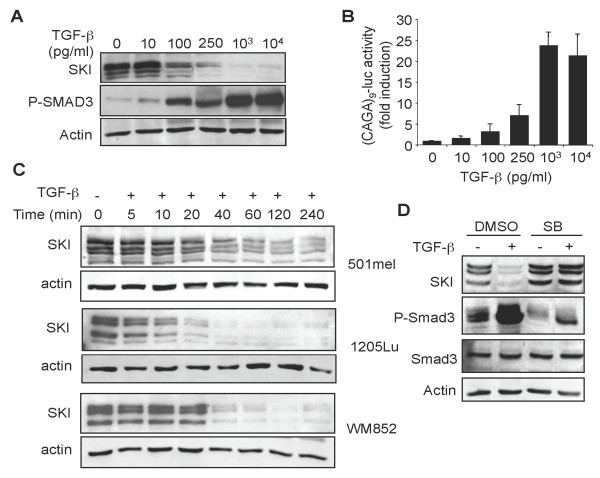
**Another High SKI expression in melanoma cells does not prevent efficient transcriptional responses to TGF-β**. A. 1205Lu melanoma cells were incubated for 60 min. with increasing concentrations of TGF-β. Cell extracts were then prepared and subjected to Western analysis for SKI and P-SMAD3 content. B. 1205Lu melanoma cells were transfected in triplicate dishes with (CAGA)_9_-MLP-luc and pRL-TK. Four hours later, TGF-β was added at the indicated concentrations and reporter activity was measured 16 h later. Results are mean+/- SD from one representative experiment. C. Three distinct human melanoma cell lines (501mel, 1205Lu and WM852) were incubated with TGF-β (10 ng/ml) for various time periods. Cell extracts were prepared and subjected to Western analysis to determine SKI content. Actin was used as an internal control. D. 1205Lu cells were incubated for 1 h in the absence (DMSO) or presence (SB) of the TβRI inhibitor SB431542 (5 μM) prior to addition of TGF-β (10 ng/ml). Protein extracts were prepared 60 min later and subjected to Western analysis for SKI content.

We next investigated whether high SKI levels in melanoma cells are associated with an absence of transcriptional responses to TGF-β. Incubation of 1205Lu melanoma cells with increasing concentrations of TGF-β for 30 min lead to a dose-dependent decrease in SKI protein content (Figure 2A), accompanied with an inversely correlated increase in P-SMAD3 levels. Parallel transient cell transfection experiments with SMAD3/4-specific (CAGA)_9_-MLP-luc reporter construct indicated dose-dependent transcriptional activation in response to TGF-β (Figure 2B).

To determine the kinetics of SKI degradation in response to TGF-β, three distinct human melanoma cell lines that exhibit high SKI protein levels in basal cell culture conditions were incubated with TGF-β; SKI protein content was monitored over time by Western blotting. Results shown in Figure 2C indicate a rapid, time-dependent, degradation of the SKI protein in all cell lines, which was abolished when cells were incubated with the TGF-β receptor type I (TβRI/ALK5) kinase inhibitor SB431542 1 h prior to TGF-β addition (Figure 2D).

In view of these experiments, it appears that despite high expression of the SKI protein, melanoma cells exhibit a strong transcriptional response to exogenous TGF-β. Rapid degradation of SKI occurs within minutes following TGF-β challenge and is accompanied with strong SMAD-dependent transcriptional activity.

Inhibition of autocrine TGF-β signaling by stable overexpression of SMAD7 in the 1205Lu cell line did not significantly alter SKI protein content, yet dramatically inhibited Matrigel™ invasion, and almost entirely blocked subcutaneous tumor growth and the appearance of experimental bone metastases in mice (Table 2), Together, these results suggest uncoupling of the pro-invasive and pro-metastatic activities of TGF-β with SKI protein levels in melanoma cells, or at least indicate that SKI function is relatively marginal as compared to the tumor promoter activities of TGF-β

**Table 2:** SMAD7 overexpression alters 1205Lu melanoma cell tumorigenicity and metastatic potential without altering SKI levels.

1205Lu clones	SKI protein levels (Western blotting)	Relative attenuation of TGF-β-dependent SMAD3/4-specific transcriptional response	Matrigel™ invasion	Subcutaneous tumor growth in mice	Bone metastases in mice
Pc	100	0	100	600	6/6 - 10/11

SMAD7a	82	70	55	nd	1/7 - 0/12

SMAD7b	107	50	45	nd	0/7 - 0/12

SKI protein levels in mock (pc) or SMAD7-overexpression 1205Lu cell populations (SMAD7a and SMAD7b) were determined by scanning densitometry of corresponding Western analyses. Matrigel™ invasion results are expressed as % of the values obtained with mock-transfected cells. Values for subcutaneous tumor growth in mice represent the average tumor volume 40 days after injection of tumor cells (mm^3^). Values for bone metastases in mice represent the ratio of mice bearing osteolytic bone metastases vs. total number of mice in each group at 5 weeks post-inoculation of tumor cells in 2 separate experiments. Details about experimental procedures may be found in [36,37]. nd: not detectable.

### Proteasome blockade prevents SKI degradation in response to TGF-β and attenuates TGF-β-driven transcriptional responses

**Figure 3:**
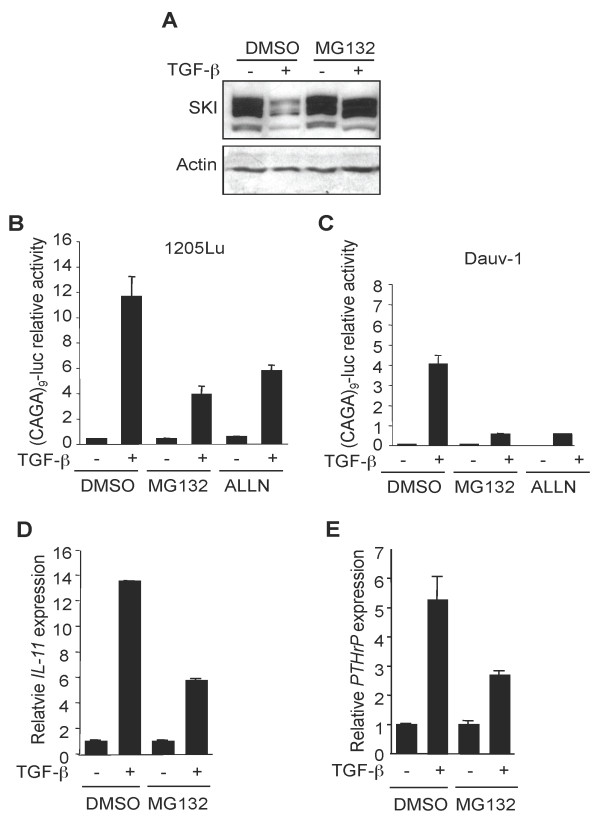
**Proteasome blockade partially antagonizes TGF-β activity and stabilizes SKI**. A. 1205Lu cells were incubated for 1 h in the absence (DMSO) or presence of the proteasome inhibitor MG132 (10 μM) prior to addition of TGF-β (10 ng/ml). Protein extracts were prepared 60 min later and subjected to Western analysis for SKI content. B, C. 1205Lu (panel B) and Dauv-1 (panel C) melanoma cells were transfected in triplicate dishes with (CAGA)_9_-MLP-luc and pRL-TK. 4 h later, cells were incubated with either DMSO, MG132 or ALLN 1 h prior to the addition of TGF-β (10 ng/ml). Reporter activity was measured 16 h later. Results are mean+/- SD from one representative experiment. D, E. 1205Lu melanoma cells were incubated with either DMSO or MG132 1 h prior to the addition of TGF-β (10 ng/ml). RNA was extracted 8 h later. *IL-11 *(panel D) and *PTHrP *(panel E) expression was measured by quantitative RT-PCR.

As expected from the literature, the proteasome inhibitor MG132 efficiently abolished TGF-β-dependent SKI degradation (Figure 3A). Also, a 1 h pre-treatment of 1205Lu and Dauv-1 melanoma cells with the proteasome inhibitors MG132 and ALLN strongly inhibited SMAD3/4-specific transcriptional response induced by TGF-β in transient cell transfection experiments with (CAGA)_9_-MLP-luc (Figures 3B and 3C, respectively). Likewise, a 1-h pre-treatment with MG132 attenuated TGF-β-induced *IL-11 *and *PTHrP *expression in 1205Lu cells (Figures 3D and 3E, respectively), two known SMAD genes targets implicated in melanoma and breast cancer metastasis to bone [36,43-45]. Thus, although SKI has little influence on TGF-β response because of its rapid degradation, it is likely that prevention of SKI degradation, as achieved by MG132 or ALLN pre-treatment of the cells, contributes to the attenuation of TGF-β-dependent transcriptional responses. This experimental approach does not however exclude that other proteasome-mediated events, independent from SKI, may also be implicated in the attenuation of TGF-β responses.

### Stable SKI knockdown in 1205Lu melanoma cells neither alters their invasive potential nor their response to TGF-β

**Figure 4:**
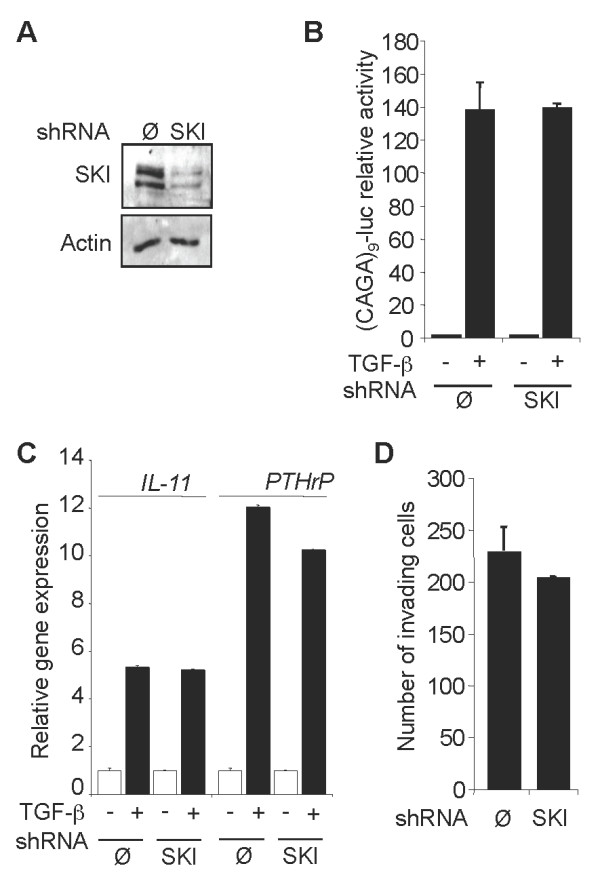
**Effects of stable SKI knockdown in 1205Lu melanoma cells**. A. Western analysis of SKI protein content in mock-transfected (Ø) and shSKI-transfected 1205Lu cells. B. Subconfluent melanoma cells were transfected with (CAGA)_9_-MLP-luc together with pRL-TK to measure transfection efficiency. Four hours post-transfection, cultures were left untreated (-) or stimulated (+) with TGF-β (10 ng/ml). Luciferase activities were measured in cell extracts 16 h post-transfection. C. Mock (Ø) and shSKI-transfected 1205Lu melanoma cells were incubated with TGF-β (10 ng/ml). RNA was extracted 8 h later. *IL-11 *and *PTHrP *expression was measured by quantitative RT-PCR. *GAPDH *mRNA levels served as internal controls. D. Comparative analysis of Matrigel™ invasion by mock- (Ø) and shSKI-transfected 1205Lu melanoma cells. Experiments were performed four times using duplicate samples.

To better understand the contribution of endogenous SKI levels to melanoma cell behavior, SKI expression was knocked down by stable expression in 1205Lu melanoma cells of a specific shRNA. Despite a 90% reduction in SKI protein content (Figure 4A), there was no significant alteration of SMAD3/4-specific transcriptional responses to TGF-β, as estimated in transient cell transfection experiments with (CAGA)_9_-MLP-luc (Figure 4B). Likewise, induction of *IL-11 *and *PTHrP *expression in response to TGF-β was not significantly altered in SKI-knockdown cells as compared to mock-transfected cells (Figure 4C). These data were further validated by means of SKI-specific siRNA transfection experiments in 1205Lu, WM852 and 888mel cells (not shown). Also, SKI knockdown did not alter the capacity of 1205Lu and WM852 (not shown) melanoma cells to invade Matrigel™ (Figure 4D).

These observations are consistent with the notion that the high levels of SKI are effectively degraded by TGF-β in these melanoma cells and therefore do not play a critical role in antagonizing, or preventing, TGF-β responses. Accordingly, we previously provided direct evidence that the invasive capacity of melanoma cells is highly dependent upon autocrine TGF-β signaling [36], further suggesting that SKI levels do not strongly influence or attenuate TGF-β effects.

### SKI knockdown fails to restore TGF-β growth inhibitory activity and p21 gene transactivation in melanoma cells

**Figure 5:**
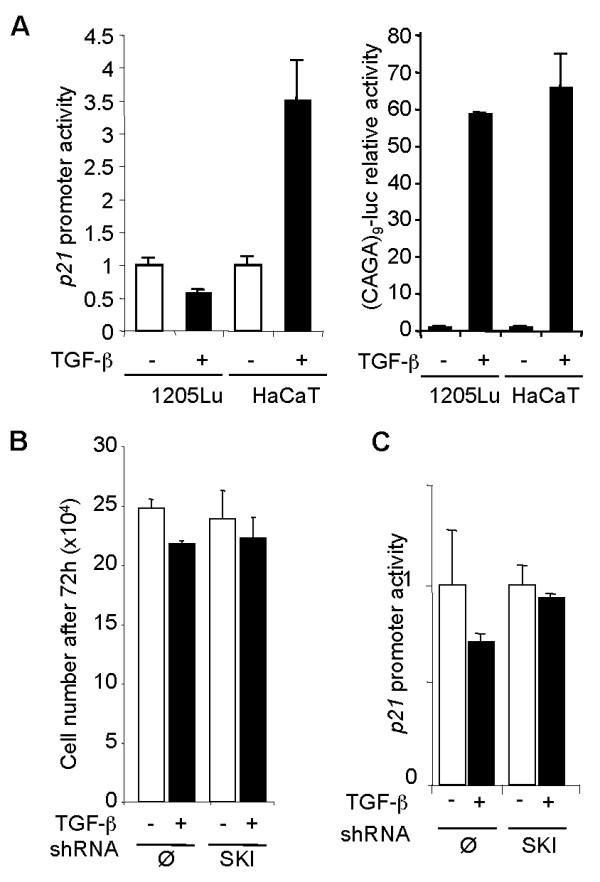
**Stable SKI knockdown in 1205Lu melanoma cells does not restore the growth inhibitory activity of TGF-β**. A. Subconfluent 1205Lu melanoma cell or HaCaT keratinocyte cultures were transfected in parallel with either a 2.4 kb *p21/WAF1 *reporter construct (left panel) or (CAGA)_9_-MLP-luc (right panel), together with pRL-TK to measure transfection efficiency. Four hours post-transfection, cultures were left untreated (-) or stimulated (+) with TGF-β (10 ng/ml). Luciferase activities were measured in cell extracts 16 h post-transfection. B. Proliferation of mock- (Ø) and shSKI-transfected 1205Lu cells in the absence (-) or presence (+) of TGF-β (10 ng/ml). C. Mock- (Ø) and shSKI-transfected 1205Lu melanoma cells were transfected in triplicate dishes with *p21*-luc and pRL-TK. TGF-β (10 ng/ml) was added 4 h later. Reporter activity was measured 16 h later. Results are mean+/- SD from one representative experiment.

It has been suggested that high SKI expression in melanoma cells is responsible for the lack of growth inhibitory activity of TGF-β, by blocking TGF-β-driven *p21 *expression [17,39]. Given the ample evidence for efficient TGF-β signaling and associated transcriptional responses in all melanoma cell lines tested thus far in our laboratory (see above and refs [35-37]), we tried to reproduce these data in the 1205Lu melanoma cell line, which is both highly invasive, strongly resistant to TGF-β growth inhibitory activity, capable of a strong SMAD3/4-specific transcriptional response to exogenous TGF-β stimulation, yet expresses high levels of SKI and SnoN proteins (see Figure 1A). Firstly, parallel transient cell transfections with either a 2.4-kb *p21/WAF1 *promoter luciferase construct or (CAGA)_9_-MLP-luc were performed in 1205Lu cells. TGF-β had no effect on *p21 *promoter activity despite efficient SMAD3/4-specific gene transcription, as measured using the highly sensitive (CAGA)_9_-MLP-luc construct (Figure 5A). As expected, *p21 *promoter transactivation in response to TGF-β was readily observed in HaCaT keratinocytes. These data confirm our initial observations that melanoma cells efficiently respond to TGF-β by a strong SMAD-specific transcriptional response [35-37], and that the lack of induction of *p21 *is highly gene-specific and is probably not due to a general inhibition of TGF-β signaling by SKI or SnoN, as SMAD3/4-specific transcription and induction of other TGF-β target genes, such as *IL-11 *or *PTHrP*, is intact. Remarkably, both the proliferative rate and the weak growth inhibition exerted by TGF-β (approx. 10% after 72 h) were virtually identical in both mock- and shSKI-transduced 1205Lu cells (Figure 5B). Also, SKI knockdown did not restore *p21 *promoter transactivation in response to TGF-β (Figure 5C). Likewise, oligonucleotide siRNA-mediated SKI knockdown in transient cell transfection experiments using 1025Lu, WM852 and 888mel cells did not allow *p21 *expression or promoter transactivation in response to TGF-β in any of those cell lines (not shown). These results are fully consistent with our previous work and with the observations provided herein that indicate that high SKI levels in melanoma cells do not antagonize the pro-tumorigenic activities exerted by TGF-β. Neither do they interfere with TGF-β-driven gene responses. It should be noted that lack of p21 induction by TGF-β in 1205Lu cells is specific, as we previously demonstrated that JNK inhibition efficiently activates p21 expression and promoter transactivation in this cell line [46].

### SKI expression in human melanocytic lesions

**Figure 6:**
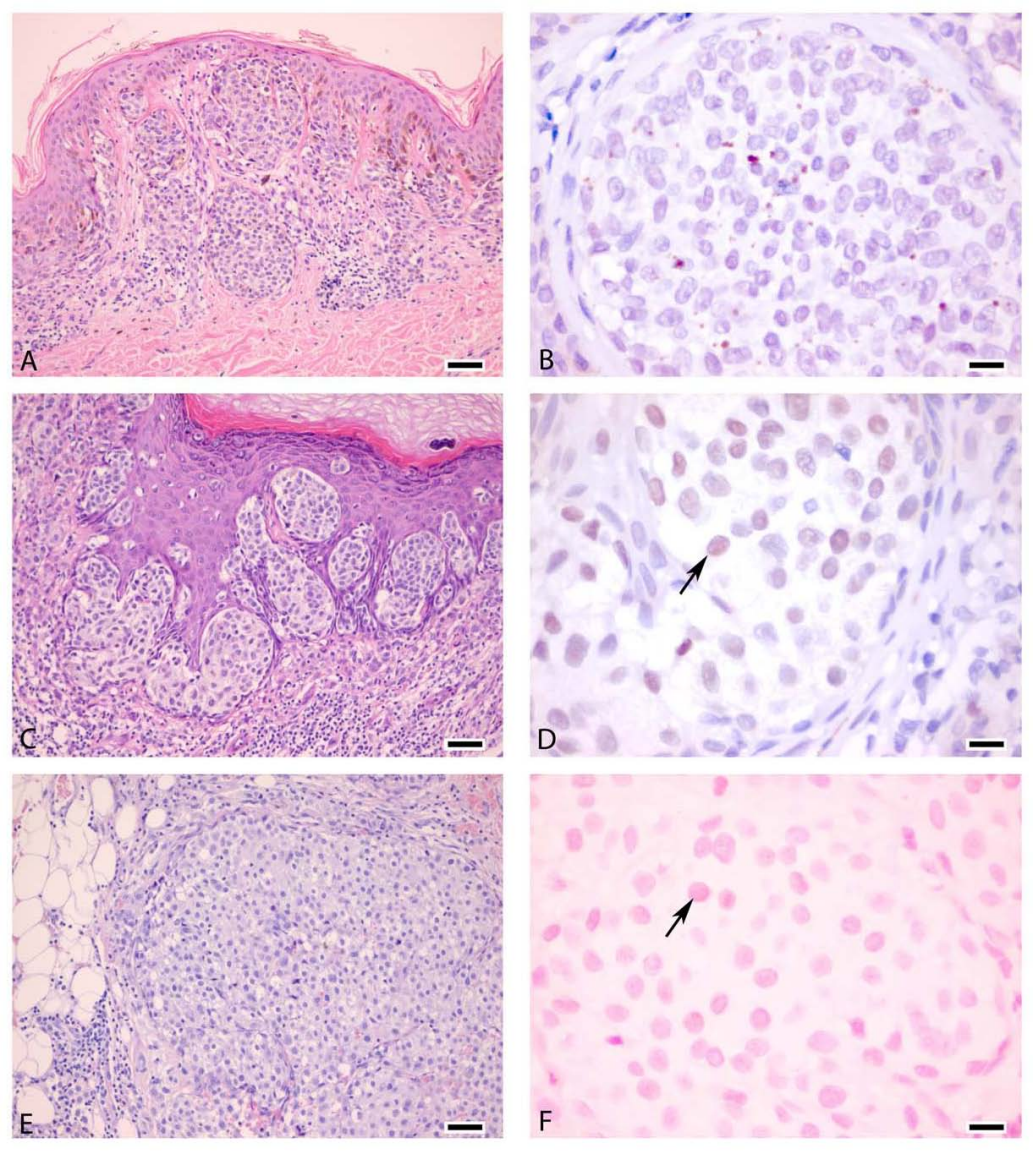
**SKI expression in melanocytic lesions**. Nevus (A, B) without nuclear staining. Clear reddish/purple nuclear staining in primary melanoma (C, D) and weak, though evident, nuclear staining in a cutaneous metastasis (A, F). Arrows in D and E indicate SKI-positive nuclei. A, C, E: H&E staining. Scale bars A, C, E: 50 μm; B, D, F: 12,5 μm.

Relatively few studies have examined the expression of SKI in melanocytic lesions in humans. We thus used immunohistochemistry to detect SKI protein in a panel of 12 nevi, 37 primary melanomas at various clinical and pathological stages of disease progression, 17 cutaneous and 10 lymph node metastases (Table 3). SKI was detected in 8 (66%) nevi, 8 (21.6%) primary melanomas, and 8 (21.7%) metastases (Table 4). Representative results for SKI staining are shown in Figure 6. We found no evidence for a link between SKI expression and histological or pathological staging within each melanoma group of samples. These data are remarkably similar to those recently reported in a larger cohort of 120 patients treated for cutaneous melanoma [47].

**Table 3:** Clinical and histopathological characteristics of a panel of human melanocytic lesions.

			Number (%)	Mean (95% CI)
**Nevi (n = 12)**				
	Gender	Male	6 (50.0%)	
		Female	6 (50.0%)	
	Age at Diagnosis (years)			36.9 (29.9-43.8)

**Primary melanoma (n = 37)**				
	Gender	Male	17 (45.9%)	
		Female	20 (54.1%)	
	Age at Diagnosis (years)			56.5 (50.3-62.6)
	Anatomic localization	Head&Neck	3 (8.1%)	
		Trunk	17 (45.9%)	
		Extremities	17 (45.9%)	
	Growth pattern	SSM	31 (83.8%)	
		NM	3 (8.1%)	
		ALM	3 (8.1%)	
	Level of invasion (Clark)	II	9 (24.3%)	
		III	9 (24.3%)	
		IV	13 (35.1%)	
		V	6 (16.2%)	
	Thickness (mm)			1.80 (1.21-2.39)

**Metastases (n = 27)**				
	Gender	Male	19 (70.4%)	
		Female	8 (29.6%)	
	Age at Diagnosis (years)			55.5 (50.5-60.6)
	Site	Cutaneous	17 (63.0%)	
		Lymph node	10 (37.0%)	

Formaldehyde-fixed and paraffin-embedded nevi (n = 12), primary cutaneous melanomas (n = 37), cutaneous and lymph node metastases (n = 17 and 10, respectively) from adult patients from the pathology archives of the Radboud University Nijmegen Medical Centre were re-evaluated by an expert pathologist. Abbreviations: SSM, superficial spreading melanoma; NM: nodular melanoma; ALM: acral lentiginous melanoma.

**Table 4:** SKI detection in a panel of human melanocytic lesions.

		SKI expression		
		Absent	Present	p-Value
**Nevi (n = 12)**		4	8	0.388^a^
**Primary melanoma (n = 37)**				
Tumor thickness	Mean (mm)	1.68	2.2	
	95% CI	1.02-2.35	0.63-3.78	0.427^b^
Level of invasion (Clark)	II	8	1	
	III	7	2	
	IV	10	3	
	V	4	2	0.779^c^
	all	29 (74.4%)	8 (21.6%)	
Growth pattern	SSM	24	7	
	NM	2	1	
	ALM	3	0	0.581^c^
Anatomical localization	Head&Neck	2	1	
	Trunk	13	4	
	Extremities	14	3	0.803^c^
**Metastases (n = 27)**				
Site	Lymph node	7	3	
	Skin	12	5	0.651^d^

Abbreviations: SSM, superficial spreading melanoma; NM: nodular melanoma; ALM: acral lentiginous melanoma. ^a^: binominal test; ^b^: Mann-Whitney U; ^c^: Pearson Chi-square; ^d^: Fisher exact test.

**Figure 7:**
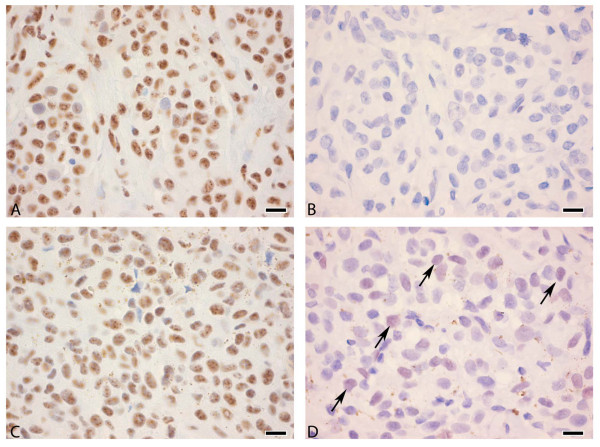
**Phospho-SMAD3C and SKI expression in melanoma**. Consecutive slides of two cutaneous melanoma metastases (A, B and C, D) were stained for phospho-SMAD3C (A, C) and SKI (B, D). The intense P-SMAD3C (A) and lack of SKI staining (B) in the first sample contrasts with the relatively low P-SMAD3C staining (C) associated with clear nuclear SKI staining (D) from the other sample, and suggests an inverse correlation between both staining intensities. Arrows in D indicate SKI-positive nuclei. Scale bars: 12,5 μm

We further analyzed the activation of TGF-β signaling in tissues by means of P-SMAD3C immunohistochemistry in a subset of melanomas and metastases. Nuclear expression of P-SMAD3C was observed in all melanocytic lesions, albeit at varying intensity (Figure 7). Intriguingly, staining intensity of SKI and phospho-SMAD3C on consecutive sections appeared to be inversely correlated (Figure 7). Although these immunohistochemical analyses do not allow quantification of protein expression, they support our observation that high TGF-β signaling can drive SKI degradation.

Taken together, the results presented herein unambiguously demonstrate (a), that SKI levels in melanoma cells are not predictive of their tumorigenic, invasive or metastatic propensity, (b), that TGF-β signals lead to rapid degradation of SKI proteins in a proteasome-dependent manner, and (c), that TGF-β induces a efficient SMAD3/4-dependent transcriptional response in melanoma cells despite high expression of c-SKI and SnoN in these cells. Furthermore, our results support the notion that there is no correlation between SKI expression and tumor progression or histogenetic subtype of human cutaneous melanomas.

## Discussion

The capacity for SKI (and SnoN) to inhibit TGF-β signaling has been extensively described. This has prompted us to consider that SKI proteins may exert tumor promoter activities, by preventing the classical growth inhibitory activity exerted by TGF-β in a variety of non-malignant cell types. Most experimental demonstrations for interference of SKI against TGF-β/SMAD signaling have largely relied on either overexpression or stabilization (by means of proteasome inhibition) of the SKI and SnoN proteins, due to the fact that TGF-β is able to rapidly induce SKI degradation in a proteasome-dependent manner [22-29]. Remarkably, in a number of neoplasms, high SKI and/or SnoN protein levels in tumor cells are observed, concomitant with (a), elevated levels of secreted TGF-β and (b), a great sensitivity of tumor cells to targeted inhibition of TGF-β signaling that strongly interferes with their tumorigenic and metastatic potential. This study was thus initiated in order to clarify the discrepancy in the literature regarding the respective roles played by TGF-β signaling and that of potentially antagonistic SKI proteins in the control of the invasive and metastatic capacities of human melanoma cells.

We, and others, have provided ample evidence that the invasive, tumorigenic and metastatic potential of melanoma cell lines is largely dependent upon autocrine TGF-β signaling. We showed initially that the SMAD cascade is activated in an autocrine fashion in a series of human melanoma cell lines [35]. We then showed that overexpression of SMAD7 in a highly invasive and metastatic cell line, 1205Lu, inhibits subcutaneous tumor growth as well as incidence and size of osteolytic bone metastases in mice, accompanied with dramatically increased survival [36,37]. Consistent with our observations, Lo and Witte [48] identified intense nuclear immunohistochemical staining of P-SMAD2 in benign nevi, melanoma *in situ*, and primary invasive melanoma, suggesting that the tumor cell autonomous TGF-β pathway is hyperactivated in response to autocrine and/or paracrine ligand activity. They demonstrated that tumor cell autonomous hyperstimulation of the TGF-β-SMAD2 pathway is causally related to melanocytic oncogenic progression in the skin and is responsible, at least in part, for the critical switch from radial to vertical growth during human melanoma histogenesis. They showed that this phenomenon requires the collaboration of activated SMADs with an altered genetic or epigenetic cellular context such as PTEN deficiency or MAPK activation [48]. Considering recent findings showing that (a) TGF-β could act of in SMAD2, SMAD3 and SMAD4-independent manner and present pro-oncogenic activity through enhancement of Ras/Raf tumorigenic transformation [49], and (b) majority of examined melanoma cells harbor activating mutation in BRAF and NRAS (BRAF V600E in WM793, 1205Lu, 983B) (NRAS Q61R in WM852), it is likely that TGF-βpromotes tumor progression through the enhancement of SKI-independent pathways, possibly MAP kinases [50]. Our data on Matrigel™ invasion support the hypothesis of uncoupling TGF-β and SKI activities.

The functional response of melanoma cells to TGF-β has been addressed by a number of laboratories. For example, it has been shown that TGF-β is a potent inducer of integrins, *IL-8*, and *VEGF *gene expression [51-54], genes implicated in metastasis and tumoral angiogenesis, respectively. A genome-wide transcriptomic analysis in over a hundred human melanoma cell lines in culture recently identified populations with very distinct gene expression profiles, the most invasive cell lines being characterized by the expression of a number of genes reminiscent of a TGF-β signature [55].

Comparable levels of expression of SKI although there is almost complete lack of the SKI protein in normal melanocytes as compared to melanoma suggest that degradation of SKI protein in normal melanocytes is far more efficient than in malignant cells and involves an alternative, yet unidentified, TGF-β-independent mechanism of SKI degradation (or translation) and that this mechanism is deregulated in melanoma cells.

The pro-metastatic role of TGF-β extends well beyond melanoma and has been extensively described in other cancers, including, but not restricted to, gliomas, breast, ovarian, colon, or prostate adenocarcinomas [44,45,56-58]. The TGF-β pathway is thus considered a prime target for preventive or therapeutic intervention in cancer [59-65]. Remarkably, Nodal, a TGF-β family member that also signals through the SMAD pathway, has been identified as playing a crucial role in melanoma progression and metastasis [66]. It is thus highly likely that increased availability of TGF-β ligands capable of activating the SMAD pathway will either bypass or overcome the inhibitory action exerted by SKI proteins, despite apparent high expression of the latter.

Our data are consistent with those reported by Medrano and co-workers [17,39] that melanoma cells in culture and human melanoma lesions exhibit high SKI protein levels. Yet, we differ significantly regarding the importance of this high of SKI in determining melanoma growth and metastasis. Our data obtained in a large panel of melanoma cell lines suggest that SKI only marginally affects TGF-β signaling: slightly elevated basal expression of some of the classical TGF-β target genes, like *PTHrP *and *IL-11*, was observed in shSKI-transfected 1205Lu melanoma cells as compared to mock-transfected cells, yet SKI knockdown only marginally affected the response to TGF-β, as estimated both at the level of target gene transcription and cell proliferation. While Reed and colleagues argued that SKI is crucial for the resistance of melanoma cells to TGF-β-induced growth inhibition and subsequent tumor growth [17,39], their data were largely obtained with the UCD-Mel-N cell line, and thus could be specific for this cell line or for a subset of melanoma cell lines, and may not be representative of all melanoma cells at large.

Noteworthy, when we initially reported that autocrine SMAD signaling occurs in melanoma cells and is dependent upon secretion and pericellular activation of TGF-β [35], we did not know the expression status of SKI and SnoN protein in the various cell lines used in our studies. In the present study, we demonstrate that autocrine TGF-β signaling is active despite high levels of SKI and SnoN protein in all melanoma cell lines (11) that we examined, including those from our initial studies. Thus, our data unambiguously demonstrate that the presence of high SKI levels is compatible with active TGF-β signaling, implying that high SKI staining in tumors may not be an indication of an absence of TGF-β-driven disease progression, as exemplified by studies with inhibitors of the TGF-β pathway that efficiently prevent melanoma tumorigenesis and metastasis [36-38]. It is possible that a subgroup of melanomas may reproduce the data obtained by Medrano and co-workers, as a similar observation was reported in a subset of esophageal carcinoma cells that are resistant to TGF-β-induced growth arrest, whereby TGF-β was unable to degrade SnoN [67].

Most critically, Chen and co-workers suggest that SKI should be considered a prime therapeutic target for melanoma treatment [39], as eliminating SKI protein would unleash the growth inhibitory activity of TGF-β. Such suggestion was recently echoed in a clinical report on the expression of SKI and SnoN in human melanoma lesion at various stages [47]. While these authors demonstrated that SKI and SnoN expression in melanoma is not associated with disease progression, they extrapolated, without experimental evidence, that SKI and SnoN may mediate the resistance of melanomas to growth inhibition by TGF-β. In our opinion, critical and potentially dangerous issues arise from the assumption that melanoma cells are not responsive to TGF-β: at advanced stages of tumor progression, therapeutic interference with invasion and metastasis, two phenomena that do not require cell proliferation and are largely under the control of TGF-β, is likely to prove essential. Targeting SKI, even though in some instance it may allow some reduction in tumor cell growth, as suggested by Medrano's group, may just do the opposite, as it would eliminate one of the natural defenses that cells have developed to interfere with autocrine TGF-β signals. Noteworthy, discrepancies about the capacity of TGF-β to degrade SKI in melanoma cells have been suggested to be due to the concentrations of TGF-β used in the various studies, and that TGF-β-induced SKI degradation only occurs at "non-physiological" concentrations [68]. This is not a satisfactory explanation as, if one follows this suggestion, increasing concentrations of TGF-β would eliminate SKI and thus exert its anti-proliferative activity and inhibit tumor progression, in contradiction with experimental evidence that inhibition of TGF-β signaling inhibits melanoma progression and metastasis. Noteworthy, given that TGF-β blockade inhibits metastasis, then whatever active concentration is present is effective to promote metastasis in spite of possible high levels of SKI expression.

## Conclusions

We provide evidence that despite high levels of c-SKI (and SnoN) oncoproteins in melanoma cells, TGF-β signaling is functional and contributes to melanoma cell invasiveness and metastasis. Exogenous TGF-β induces a rapid, proteasome-mediated, degradation of c-SKI, not accompanied by an inhibitory activity of TGF-β on melanoma cell proliferation. While understanding the exact role played by labile c-SKI protein in melanoma remains to be understood, we believe that targeting SKI to prevent tumor spreading and disease progression is likely not an appropriate therapeutic strategy.

## Methods

### Cells, plasmids and reagents

Melanoma cell lines (1205Lu, WM852, WM983B, SK28, MNT1, Dauv-1, Fo-1, WM239A, WM1341D, SK-mel501, SK-mel888) have been described previously [35,42,69-71]. NHEM (Normal Human Epidermal Melanocytes) were purchased from Promocell (Heidelberg, Germany) and cultured in ready-to-use medium, also supplied by Promocell. All cells were grown at 37°C in a humidified atmosphere of 5% CO2. The reporter plasmids (CAGA)_9_-MLP-luc [72] and 2.4 kb p21/WAF1 promoter luciferase reporter construct [73] were gifts from Drs. Sylviane Dennler (INSERM U697, Paris, France) and Bert Vogelstein (Johns Hopkins University, Baltimore, MD), respectively. The pRL-TK vector was from Promega (Madison, WI). pSuper vector expressing *SKI *shRNA has been described previously [22]. Human recombinant TGF-β1, purchased from R&D System Inc. (Minneapolis, MN) is referred to as TGF-β throughout the text. The ALK5/TβRI inhibitor SB431542, and the proteasome inhibitor leu-leu-leu-al (MG132) were both from Sigma-Aldrich (St. Louis, MO). Acetyl-leu-leu-norleu-al (ALLN) was purchased from CalBiochem (San Diego, CA).

### Immunoblotting experiments

Protein extraction and Western blotting were performed as previously described [74]. Anti-SMAD3 and anti-Actin antibodies were purchased from Zymed (San Francisco, CA) and Sigma-Aldrich, respectively. The rabbit anti-phospho-SMAD3 antibody [75] was a generous gift from Dr. Edward Leof (Mayo Clinic College of Medicine, Rochester, MN). Anti-c-SKI, anti-SnoN, anti-SMURF2, anti-HSP60 and secondary anti-mouse and anti-rabbit horseradish peroxidase-conjugated antibodies were from Santa Cruz Biotechnology Inc. (Santa Cruz, CA). Anti-Arkadia was obtained from Abnova Gmbh (Heidelberg, Germany).

### Cell transfections and luciferase assays

Melanoma cells were seeded in 24-well plates and transfected at approximately 80% confluency with the polycationic compound Fugene (Roche Diagnostics, Indianapolis, IN) in fresh medium containing 1% FCS. TGF-β and/or inhibitors were added 4 h after transfections. Following a 16-h incubation, cells were rinsed twice with PBS and lysed in passive lysis buffer (Promega). pRL-TK (Promega) was co-transfected to assess transfection efficiency. Luciferase activities were determined with a Dual-Glo luciferase assay kit according to the manufacturer's protocol (Promega). Each experiment was repeated at least three times using triplicate dishes in each of them for each experimental condition.

### RNA extraction and gene expression analysis

Total RNA was isolated using an RNeasy™ kit (Qiagen GmbH, Hilden Germany). Genomic DNA contaminations were eliminated by DNAse I treatment. One microgram of RNA from each sample was reverse transcribed using the Thermoscript kit (InVitrogen) following the manufacturer's protocol. The resulting cDNAs were then processed for either semi-quantitative or real-time PCR using SYBR Green technology. In the latter case, reactions were carried out in a 7300 Real-time PCR System (Applied Biosystem) for 40 cycles (95°C for 15 sec/60°C for 1 min) after an initial 10-min incubation at 95°C, using the following primer sets: *SKI *(sense, 5'-gagaaattcgactatggcaacaag-3'; antisense, 5'-gtcatctgttttgggtcttatgga-3'; *IL-11 *(sense, 5'-actgctgctgctgaagactc-3'; antisense, 5'-ccacccctgctcctgaaata-3'); *PTHrP *(sense, 5'-tttacggcgacgattcttcc-3'; antisense, 5'-ttcttcccaggtgtcttgag-3'); *GAPDH *(sense, 5'-gctcctcctgttcgacagtca-3'; antisense, 5'-accttccccatggtgtctga-3'). Data were analyzed using Applied Biosystems Sequence Detection Software (version 1.2.1).

### Matrigel™ invasion assays

Tissue culture Transwell^® ^inserts (8-μm pore size, Falcon, Franklin Lakes, NJ) were coated for 3 h with 10 μg of growth factor-reduced Matrigel™ (Biocoat, BD Biosciences, San Jose, CA) in 100 μl of PBS at 37°C. After air-drying the chambers for 16 h, the Matrigel™ barrier was reconstituted with 100 μl DMEM for 24 h at 37°C. The chambers were then placed into 24-well dishes containing 750 μl of W489 medium supplemented with 0,1% FCS. Cells (5 × 10^4^) were added to the upper well of each chamber in 500 μl of serum-free W489 medium. After a 24 h-incubation period, the number of invading cells was counted by bright-field microscopy at ×200 in six random fields. Additional details of the procedure may be found in [37].

### Cell proliferation assays

Melanoma cells were plated in 12-well plates at an initial density of 5000 cells/well. Cell growth was measured after 72 h in 1% FCS, with or without TGF-β (10 ng/ml), by counting the cells after trypsinization using a Malassez cell. For each experimental condition, duplicate dishes were counted. Experiments were performed at least three times with similar results.

### Human tissues

Formaldehyde-fixed and paraffin-embedded naevi (n = 12), primary cutaneous melanomas (n = 37), cutaneous and lymph node metastases (n = 17 and 10, respectively) from adult patients were obtained from the pathology archives of the Radboud University Nijmegen Medical Centre, and re-evaluated by an expert pathologist (see corresponding Results section for details). Tissues were obtained according to local ethical guidelines and approved by the local regulatory committee.

### Immunohistochemistry

Paraffin-embedded 4 μm sections on superfrost slides (Menzel-Glaser, Braunscheig, Germany) were de-waxed in xylene, rehydrated through graded alcohol baths, then rinsed with PBS. After quenching of endogenous peroxidases, an antigen retrieval step (sodium citrate 10 mM, pH 6.0 for 10 min. at 95°C) was performed. Tissue sections were subsequently pre-incubated with 20% normal goat serum in PBS, followed by an overnight incubation with rabbit polyclonal SKI antibody (H-329, 1 μg/ml, Santa Cruz Biotechnology Inc.) or affinity-purified rabbit polyclonal anti-phospho-Smad3C (P-SMAD3C) antibody [75] in PBS containing 1% bovine serum albumin overnight at 4°C. For detection of SKI, the Powervision system (Immunovision Labs, Brisbane, CA) was used as a secondary reagent with 3-amino-9-thylcarbazole served as a chromogen. For detection of P-SMAD3C, a biotin-avidin peroxidase complex was generated according to standard procedures (Vector, Burlingame, CA) and developed with 3,3'-diaminobenzidine. Counterstaining was performed with hematoxylin. Samples with nuclear SKI appear purple and were scored positive in case positivity was detected in at least 10% of melanocytic cells.

### Statistical analyses

Data were entered in a computerized database and analyzed using SPSS software (version 16.0.1 for Windows). The binomial test was used to analyze frequency of SKI expression in nevi. The Mann-Whitney U test was used to correlate SKI expression and tumor thickness. The correlation between SKI expression and the level of invasion was determined by the Pearson Chi-square test. Fisher exact test for small sample numbers was used to determine the correlation between SKI expression in cutaneous and nodal metastases.

## Competing interests

The authors declare that they have no competing interests.

## Authors' contributions

DJ, VIA and ELS performed gene expression and protein studies in vitro. LVK performed the immunohistochemical studies on human samples as well as statistical analyses. KL and AM contributed to the design of the study and drafted the manuscript. All authors read and approved the manuscript.
